# Pigment Epithelia of the Eye: Cell-Type Conversion in Regeneration and Disease

**DOI:** 10.3390/life12030382

**Published:** 2022-03-06

**Authors:** Eleonora N. Grigoryan

**Affiliations:** Kol’tsov Institute of Developmental Biology, Russian Academy of Sciences, 119334 Moscow, Russia; leonore@mail.ru; Tel.: +7-(499)-1350052

**Keywords:** eye, pigment epithelia, cell-type conversion, regeneration, disease, molecular mechanisms

## Abstract

Pigment epithelial cells (PECs) of the retina (RPE), ciliary body, and iris (IPE) are capable of altering their phenotype. The main pathway of phenotypic switching of eye PECs in vertebrates and humans in vivo and/or in vitro is neural/retinal. Besides, cells of amphibian IPE give rise to the lens and its derivatives, while mammalian and human RPE can be converted along the mesenchymal pathway. The PECs’ capability of conversion in vivo underlies the lens and retinal regeneration in lower vertebrates and retinal diseases such as proliferative vitreoretinopathy and fibrosis in mammals and humans. The present review considers these processes studied in vitro and in vivo in animal models and in humans. The molecular basis of conversion strategies in PECs is elucidated. Being predetermined onto- and phylogenetically, it includes a species-specific molecular context, differential expression of transcription factors, signaling pathways, and epigenomic changes. The accumulated knowledge regarding the mechanisms of PECs phenotypic switching allows the development of approaches to specified conversion for many purposes: obtaining cells for transplantation, creating conditions to stimulate natural regeneration of the retina and the lens, blocking undesirable conversions associated with eye pathology, and finding molecular markers of pathology to be targets of therapy.

## 1. Introduction

**Figure 1:**
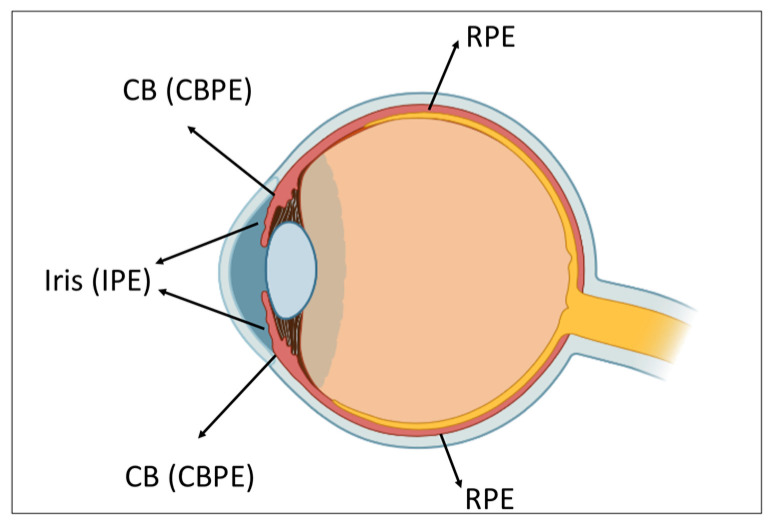
Pigment epithelia in the structure of the eye of vertebrates. RPE—retinal pigment epithelium; CB—ciliary body, CBPE—Pigment epithelium of ciliary body; IPE—iris pigment epithelium.

The pigment epithelial cells (PECs) in the vertebrate eye are considered both a potential source of eyes tissues regeneration and a cause of a number of eye disorders. The cells of retinal pigment epithelium (RPE), ciliary body pigment epithelium (CBPE), and iris pigment epithelium (IPE) are distinguished by the capability of altering their phenotype (Figure 1). Therefore, the analysis of the conversion specifics that underlie both the biology of these cells and the mechanisms of regulation of cell differentiation is of particular value.

The term “cell-type conversion”, concerning a process that occurs both in vivo and in vitro, means an alteration of somatic cells’ identity, a change in the differentiation type, and is also referred to as transdifferentiation [1,2,3]. Currently, the terms “cell-type conversion”, “transdifferentiation”, and “cell-type switching” are used in various contexts depending on changes in phenotypes of various cells, ranging from fully differentiated and specialized to stem cells. Cell-type conversion can be much more frequently observed in vitro than in a live organism where conditions are known to rigidly stabilize the terminal cell differentiation. The in vitro cell conversion described in the literature is achieved by its induction in targeted experiments with the exposure of cells to transcription factors (TFs), regulatory signaling molecules, and also with changes in the physicochemical properties of culture substrates. The induced pluripotent stem cells (iPSCs) obtained from cultured embryonic and mature mouse fibroblasts in vitro under conditions of overexpression of such TFs as Oct4, Sox2, Klf4, and c-myc (OSKM) in cells is the most commonly known example [4]. A less known one is the induction of cell-type conversion in vitro using the TF MyoD. Transfections of the *MyoD* gene contribute to the transformation of RPE cells and also mammalian fibroblasts and chondrocytes into muscle cells [5,6]. In a culture of chick RPE cells, the TF *Sox2* promotes the expression of the retinal ganglion and amacrine cell markers [7]. The use of signaling regulatory molecules is a mandatory attribute of the cell–phenotype switching induction during cell cultivation. Thus, e.g., FGF2, TGF-beta, and Wnt are widely known as stimulators of RPE conversion in vitro [8,9,10,11,12].

Natural cell-type conversion in vertebrates in vivo also provides ample examples. Besides those related to pigment epithelia of the eye, which are considered in detail in the present review, the modes of natural cell-type conversion in vivo are diverse as regards the initial cell type, the eventual result of the process, and also the class of organisms in which the phenomenon is observed. The known examples for mammals are as follows: the appearance of hepatic foci in the pancreas, the development of intestinal tissue at the lower end of the esophagus, and the formation of muscle cells, chondrocytes, and neurons from neural precursor cells (reviews: [13,14,15]). Considering these examples is beyond the scope of the present review, where major focus is on the pigment epithelia of the eye.

Having similar ectodermal origins, pigment epithelia of the vertebrate eye—IPE, CBPE, and RPE—are examples of cell-type conversions in vertebrates in vivo and in vitro. The phenomenon has been shown and studied for members of various vertebrate classes including humans. The IPE cells are capable of phenotype conversion in some fish and amphibian species in vivo, and also in amphibians, birds, and mammals in vitro. Natural conversion of RPE cells was found not only in amphibian and avian, but also mammalian embryos. The RPE cell conversion in vitro can be revealed by culturing cells of both embryos and adults from different vertebrate classes, including humans.

It is important to note that there are differences in the pathways of conversion chosen by different animals and its outputs, resulting in the type of new differentiation acquired. The known examples are conversions of eye’s pigment tissues into lens cells, retinal cells, glial cells, and also cells of the mesenchymal spectrum of differentiation. All of these examples are discussed in detail in the respective chapters of the review. It should also be noted that pigment epithelia in the process of transdifferentiation in vivo passes through a transitory, “dedifferentiated” state, necessary for the loss of the original phenotypic traits by cells, and also through the proliferation and manifestation of traits of another cell type. The dedifferentiation stage includes dynamic reprogramming of the genome and epigenome and, as a result, alterations in the cell morphology and metabolome. During the conversion, at the stage of transition from one differentiation to another, some specific properties of the original cells, traits of multipotency, and certain traits of the new emerging differentiation may coexist (reviews: [3,16,17]).

The phenomenon of cell-type conversion with the acquisition of a new identity by cells is directly associated with two key issues in ophthalmology: the eye tissue regeneration and the treatment of eye tissue pathologies in the case of retinal disorders associated with cell-type conversion. In mammals and humans, retinal detachment and rupture are known to lead to RPE cell conversion along the mesenchymal pathway, and to diseases such as proliferative retinopathy [18,19,20], proliferative diabetic retinopathy [21], and subretinal fibrosis [22]. Currently, the potential of the retinal regeneration from endogenous cell sources related to eye’s pigment epithelia is being widely studied and discussed (reviews: [23,24,25,26,27,28]).

The study and understanding of the IPE, CBPE, and RPE conversion along the neural/retinal pathway are crucial to stimulate the process for providing cell replacement in the damaged retina. Endogenous cell sources for eye retina repair are an important and attractive alternative to the use of cells derived from induced pluripotent stem or embryonic cells (iPSCs, ESCs) [29,30,31].

The review considers the course of conversion of pigment epithelia in vertebrates and humans, and also their comprehensive, dynamic molecular–genetic and epigenetic regulation. An attempt has been made to identify the initial properties of gene expression and epigenome, conditioned by phylo- and ontogenetic development, that determine the conversion of pigment epithelia of the eye, and also the similarities/differences in the patterns of conversion and the methods to regulate and control it. The analysis of the conversion of different pigment epithelia, having, however, a common origin and similar phenotypes, makes it possible to understand the factors that dictate a particular cell-type conversion strategy.

The interest in the phenomenon of cell-type conversion and the further development of knowledge about it are relevant for many reasons. These are required to understand to what extent the mechanisms of this process are common and what regulatory mechanisms provide similarities and differences between the positive (regeneration) and negative (pathology) results of transdifferentiation of the same tissue in different vertebrates. This knowledge is of practical value to be potentially applied in regenerative medicine for the purpose of protecting eye tissues, in particular the retina, from degenerative disorders and creating conditions for regeneration. The resulting data help in modeling a number of diseases associated with pigment epithelium conversion and serves to prevent uncontrolled transformations of eye tissues accompanied by active cell proliferation and posing a risk to human health. At last, the identification of key molecules and links in the regulatory mechanisms of cell-type conversion identifies the targets and factors influencing them, which allows prevention and treatment of pathology.

## 2. Cell-Type Conversion of Iris Pigment Epithelial (IPE) Cells

### 2.1. Cell-Type Conversion of IPE Cells In Vivo

The iris plays an important role in the visual function of the eye. In vertebrates and humans, it regulates the flux of incoming light and serves for the focal adjustment of closer objects. The iris is included in the circulation of the aqueous humor and is, thus, involved in the regulation of intraocular pressure [32]. During embryogenesis, the iris arises from both the optic cup and the periocular mesenchyme [33], which may affect IPE’s capability of cell-type conversion in vitro and in vivo. Despite the structural and functional specialization, IPE demonstrates, as shown below, the ability to convert into RPE cells, lens cells, and retinal neurons.

In a number of caudate amphibians [34,35,36,37,38], some fish species [39], and avian embryos [40,41,42], the lost lens can regenerate through the conversion of IPE cells into lens fibers. For mammals, the lens regeneration was reported for mice [43]. The authors caused the lens to be formed in 7 day-old and adult mice by stimulating regeneration with intraperitoneal injections of retinoic acid (RA). The resulting regenerates were identical to the normal organs in their morphological properties.

**Figure 2:**
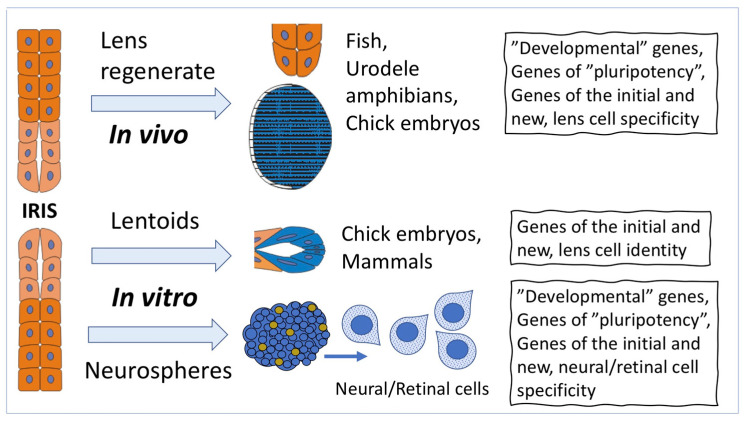
Types of iris pigment epithelium cells’ (IPECs) conversion. On the right—the main groups of genes expressed during lens regeneration in vivo, in lens-like structures (lentoids)’ originated from IPE iris in tissue culture, and in neural/retinal cells emerged from of IPECs-derived spheroids in vitro (right). More details are in the text.

The Wolffian lens regeneration from IPE cells in newts (Urodela, Salamandridae) is a classic example of IPE cell-type conversion (Figure 2). The process has been studied comprehensively, from the morphology of certain stages to their molecular mechanisms [2,38,44,45,46,47,48]. The organization of the cytoskeleton of IPE cells in newts allows attributing them to the myoepithelial type [49]. After removing the lens in newts, the cells of the pupillary margin in the dorsal region of IPE disconnect, lose their original specific traits, remodel the cytoskeleton, proliferate, and produce a population of dedifferentiated descendant cells that are a source of the lens regenerate (lens vesicle) to form. The latter, multiplying the number of cells, grows, undergoes morphogenesis, forms lens epithelium and nucleus, and then is separated from its source, IPE. One of the trigger signals to recovery after the lens removal is the expression of thrombin in the distal part of the dorsal region of the iris, the site of lens regeneration [50]. Leukocytes, attracted by the fibrin clot containing thrombin and the transmembrane protein tissue factor (clotting factor III), activate the expression of FGF2, which, in turn, induces the entry of IPE cells into the cell cycle [51]. During the lens regeneration from IPE cells, FGF2 is a key regulatory factor whose role is shown to be essential during the reprogramming of IPE cells and at the proliferative stage of lens regeneration. FGF2 controls the process through the up-regulation of a number of genes associated with cell cycle regulators, cytoskeleton alterations, and transcription [52,53,54]. Besides FGF2, other signaling pathways—BMP, Wnt, Retinoids, and their receptors—have been shown to also play their roles in lens regeneration [55,56]. The significant similarity of the signaling pathways that regulate the regeneration of the lens and its development in ontogenesis is emphasized in discussions [55].

IPE-derived dedifferentiated cells of the lens vesicle have been compared to tissue stem cells [47,57]. This comparison has a reason. During the dedifferentiation after lentectomy, IPE cells pass through a stage where traits of early progenitor cells are recorded. This conclusion is made not only on the basis of morphological patterns and the proliferative activity of IPE cells, along with the inhibition of melanin synthesis in IPE cells [58], but also on the basis of the data showing the initiation of TFs’ expression from a number of pluripotency inducers such as *Sox2*, *Klf4*, and *c-myc* [59]. The same model—cells of the developing IPE-derived lens regenerate—showed the expression of the histone B4 that is characteristic of early oogenesis and a change in the ratio of histones B4/H1 toward B4 [60]. Another reported observation is the expression of the nucleostemin (Ns) gene and protein and the accumulation of the latter in nuclei of IPE cells undergoing conversion [61]. A similar phenomenon has been described from mammalian stem and tumor cells (Tsai, McKey, 2002) [62]. During the active proliferation of IPE cells in situ, in parallel with dedifferentiation, there is neither uncontrolled growth nor disturbance of the lens’ regeneration and morphogenesis, even in the case of repeated lens removals [63]. The rare cases of tumor formation are shown to be associated with exposure to carcinogens [64] and also occur in newts exposed to low gravity conditions [65].

The common ectodermal origin of the eye retina, iris, and lens probably explains the cases of formation of lentoids, the lens-like structures synthesizing crystallins, in tissue cultures from the retina and iris of birds [66] and rodents [67] (Figure 2). Currently, lentoid bodies expressing crystallins are obtained from human embryonic (hESC) and induced human (iPSC) stem cells [68,69]. An ultrastructure analysis of hESC- and iPSC-derived lentoid bodies has identified closely packed lens epithelial and differentiating fiber cells. A comparison of the mouse lens epithelial and fiber cell transcriptomes with hESC- and iPSC-derived lentoid body transcriptomes has shown a more then 96% overlap [69].

### 2.2. Cell-Type Conversion of IPE Cells In Vitro

IPE has a common origin and some similarity with RPE: it has an apical–basal axis of polarity, tight junctions, microvilli, and contains pigment in cytoplasm. IPE cells cultured in vitro have shown a potential to carry out many functions characteristic of RPE cells, e.g., specific phagocytosis of rod outer segments and retinol metabolism. These observations have served as a basis for an attempt to accumulate IPE-derived cells in vitro for subsequent transplantation in pathologies associated with RPE cells’ death [70].

Of major interest, however, is the IPE cells’ capability of conversion into neural cell-types which could be used for transplantation in case of degenerative retinal diseases in humans without gene transfer [71] (Figure 2). The data on the isolated chick IPE cells (2 days post hatching) that formed spheres containing cells with proliferative potential and expressing TFs, which are markers of cell progenitors, gave some hope. After transferring the spheres to laminin, cells of neural phenotypes resembling retinal ones were detected. When co-cultured with the chicken embryonic retina, these cells exhibited the properties of photoreceptors and Müller glial cells. In contrast, in adherent culture on collagen-coated dishes, IPE-derived progenitors could revert to the pigment epithelial phenotype, indicating the reversible state of IPE-derived cells during reprogramming [71]. Recent studies on the same model, chick eye IPE in vitro, have indicated the role of the Wnt signaling pathway in the negative regulation of the process of IPE cell conversion along the neuronal pathway [72].

Isolated iris cells of postnatal and adult mice also exhibit properties of stem-like cells in vitro: the expression of nestin and Pax6 [73]. IPE cells of adult rats in vitro can be reprogrammed along the neuronal/retinal pathway, producing photoreceptor-like cells. This occurs in the presence of FGF2 in the medium [74]. In the case of adult pig IPE cells cultured in vitro, the appearance of proneural cell progenitors with the ability to differentiate into photoreceptor-like cells was recorded. The process was initiated without the involvement of fetal serum and growth factors, but the presence of FGF2 and IGF2 in the medium significantly stimulated their conversion [12].

In a comparative study of IPE-derived progenitor cells, cultured IPE cells of adult rats and primates proved to exhibit similar characteristics of photoreceptor phenotypes [75]. The cell phenotypes composing the spheres obtained in vitro from human IPE cells are not an exception. They exhibit proliferative activity and express markers of progenitors such as Sox2, Nanog, nestin, and also GFAP. However, it is noted that many cells retain the properties of differentiated epithelial cells and lack the key properties of stem cells [76]. The study by Seko et al. [77] has demonstrated that iris cells of human infants in vitro express stem cell markers such as Nestin, N-cadherin, SOX2, Musashi-1, and PAX6. To produce light-responsive photoreceptor-like cells, Seko and co-authors used combinations of “developmental” TFs. Expression of such photoreceptor molecules as rhodopsin, blue opsin, and green/red opsin in induced photoreceptor cells were dependent on TFs’ combinations: *CRX* and *NEUROD* induced rhodopsin and blue opsin, but did not induce green opsin; a combination of *CRX* and *RX* induced blue opsin and green/red opsin, but did not induce rhodopsin. Phototransduction-related genes, as well as opsin genes, were up-regulated in those cells. The combination of *CRX* and *RX* generated immature photoreceptors, and additional *NEUROD* promoted maturation. These data suggest that photosensitive photoreceptor cells can be generated through manipulations with TF combinations [77]. To continue studying the potential of mammalian IPE cells’ conversion along the retinal pathway, Bennis et al. [78] compared the expression profiles of human RPE and IPE genes. An analysis of transcriptomes has indicated significant molecular similarities, but, nevertheless, revealed some differences. The latter included, along with the enriched RPE gene expression, prominent features which were involved in the phototransduction cascade. Differences were also found in the activity of the Wnt signaling pathway: it was active in IPE, but not in RPE. However, when considered together, the data suggest that both IPE and RPE cells retain developmental or functional plasticity and can serve to develop strategies for replacement of lens and retinal cells [78]. In the work based on human IPE in vitro, Yamamoto et al. [79] identified cells that were positive for p75NTR, a marker of retinal tissue stem and progenitor cells. The p75NTR^+^H-iris cells, selected by sorting, expressed markers of retinal neurons. Among them, recoverin-positive cells and photoreceptor-like cells with electrophysiological functions were isolated and accumulated. Retinal ganglion cells with electrophysiological functions were also differentiated by changing the culture method [79].

As can be seen from the above information, the IPE cell-type conversion process includes a stage where reversible and incomplete inhibition of the expression of genes responsible for IPE specialization is assumed, which occurs along with the initiation of the expression of genes that determine a new differentiation. This assumption, previously made for amphibian IPE [3], is now confirmed for both IPE and other pigment epithelia of the vertebrate eye, RPE and CBPE [16,17]. The above observations also indicate that the expression of principal genes characteristic for stem/progenitor cells occurs during reprogramming. This information allows raising and answering the main question as to how and at what point the desired IPE cells conversion pathway should not only be set, but also fixed experimentally. This will make it possible to use IPE-derived cells with a specified and stable in vitro differentiation for the transplantation of the obtained cells in retinal pathologies. The development of approaches to the transplantation of autologous IPE-derived cells for the treatment of degenerative diseases of the human retina is underway [80,81].

Among the congenital and acquired diseases of the human iris proper, those associated with iris cell-type conversion have not been reported to date [82]. The iridocorneal fibroplasias leading to iridal–corneal adhesion may be remotely associated with cell conversion along the mesenchymal pathway. Adhesion may be congenital or acquired and may cause opacities in either the cornea or the lens. Spontaneous adhesions are observed in albino rats, but are rare in mice [83]. These changes are assumed to be associated with the production of fibrils, which are ECM molecules synthesized by the transforming IPE-derived cells that have acquired the properties of mesenchymal differentiation. However, fibrotic changes in the lens capsule cells may also be, to an equal extent, a cause of the accumulation of mesenchymal fibers between the lens and the iris [84].

The spectrum of cell-type conversions for IPE cells in pathology includes cases of uveal melanomas, which is a rather rare malignant transformation of these cells in humans [85]. Neoplastic degenerations of the eye PECs, having their own epidemiological, clinical, physiopathological, and molecular features, as well as mechanisms involved in metastasis, are beyond of the scope of the present review. Also, the cases of the formation of iris cysts [86] are not considered in the review’s discussion. Primary cysts arise either from the iris pigment epithelium or from the iris stroma. The potential of iris stroma cells’ conversion into neuronal cells was also discussed in the above-cited study on cultured pig IPE cells [12].

## 3. Cell-Type Conversion of Ciliary Body Epithelial Cells

### 3.1. Cell-Type Conversion of CB Cells In Vivo

The mammalian ciliary body (CB), located between *ora serrata* of the neural retina (NR) and the iris, has an anatomical homology with the ciliary marginal zone (CMZ) of fish and amphibians, an undifferentiated region known as a source of new neurons and glial cells of the retina in development and regeneration (reviews: [28,87,88]). However, Miles and Tropepe [88] insist not to confuse the CB region with the ciliary, non-stratified region of the retina, which contains low-differentiated cells in vertebrates.

The mammalian CB, unlike the fish and amphibian CMZ, is a highly specialized and multifunctional eye structure [89,90]. It includes ciliary muscles and two folded epithelial layers: unpigmented and pigmented (ciliary body pigment epithelium, CBPE). The former is a continuation of the ciliary NR region; the latter is a continuation of RPE. CB is known to be responsible for secreting the aqueous humor in the anterior chamber and the vitreous humor and protein components, including crystallin, Optc (opticin), collagen, and laminin proteins [91,92]. The CB muscles, supporting the iris, are involved in the lens accommodation along with the latter. A mass spectrometry study of the CB proteome in humans has revealed a wide range of specific proteins that reflect CB functions [93]. The profile of gene expression in the cells of pigment and non-pigment epithelial layers of the human CB was studied by microarray [94]. The observed similarity of both layers, in turn, indicated the similar functions performed by them. The differences were in the specifics of cell phenotypes. CBPE cells expressed pigment cell-specific genes involved in pigmentation, cytoskeleton, endocrine and metabolic pathways, and NR developmental properties. With respect to them, it was found that the pathway of “human embryonic stem cell pluripotency” and Wnt/β-catenin signaling differed significantly between the non-pigment and pigment epithelia of CB [94]. Differences were also found between the two CB layers on the level of expression of a number of markers: P-Cad (Cdh3) is expressed in CBPE, while the expression of N-Cad (Cdh2), Zic2, and Chx10 occurs in the non-pigment layer [95]. It is important that, in the analysis of certain cell types, the CBPE cells that demonstrated the expression of P-Cadherin were the source of the neurogenic potential in vitro. Nevertheless, there was an ambiguity in the question as to how much the P-Cadherin expression is required to maintain CBPE cells’ “stemness” in vivo and for the formation of the clonal stem cell spheres in vitro [95].

Studies of CB cell conversion in vivo showed that the proliferation was activated, and that the phenotype of CB cells changed in response to the optic nerve axotomy, which caused the death of retinal ganglion cells in adult mice. The subpopulation of those cells was positively stained for homeodomain protein Chx10 and recoverin, a marker for photoreceptors and bipolar cells in the retina [96]. When studying the molecular mechanisms responsible for the regulation of proliferation and progenitor genes’ expression in adult mouse CB cells, Del Debbio et al. [97] performed intraocular injections of activity regulators of Rho GTPases, which are participants in the regulation of signaling pathways that control cell proliferation and transcription. Activation of Rho GTPases increased the co-expression of Pax6 and Chx10, but had no significant effect on the proliferation of CBPE. In contrast, the Rho GTPases’ inactivation increased cell proliferation and potentiated the proliferative effect of growth factors. The authors suggest that the modulation of CB cells’ proliferation and reprogramming may provide a potentially new approach to retinal repair [97].

### 3.2. Cell-Type Conversion of CB Cells In Vitro

When compared to in vivo studies, in vitro experiments provide much more extensive information about the conversion capabilities of CB cells. In studies of the initial proliferative potential of mammalian eye CB cells, Coles et al. [98] used mutant mice with abnormalities of eye development, in particular, RPE. *Mitf^mi/mi^* mutation caused complete loss of differentiated RPE population, while *Chx10^orJ/orJ^* mutant mice had a small eye phenotype, characterized by a reduction in proliferation of the neural retina progenitors, a loss of mature bipolar cells, and a severely hypocellular neural retina in adulthood. In adult mice with these eye development abnormalities caused by mutations in two “developmental” genes, CB cells showed higher proliferative activity with three to eight-fold increased retinal stem cell population when compared to wild-type control eye. Cells isolated from the CB of *Mitf^mi/mi^* mice and exposed to in vitro conditions during sphere formation had the ability to differentiate to neural cell types including photoreceptor and glial lineages. CB-derived sphere cells of *Chx10^orJ/orJ^* mice gave rise to Müller glia and neural cell types including ganglion, amacrine cells, and photoreceptors [98]. Thus, a direct correlation was found between the proliferative activity and cell-type switching of CB cells.

**Figure 3:**
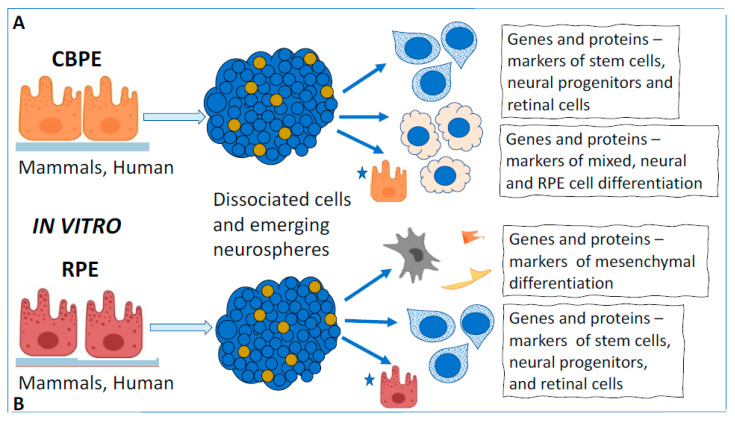
Cell-type conversion of pigment epithelial cells of ciliary body (CBPE) (**A**) and retina (RPE) (**B**) under conditions of cell culture in vitro. Asterisk—cells of CBPE and RPE that maintain their original differentiation. More details are in the text.

Numerous studies have shown that human, monkey, pig, rodent, and chicken CBPE cells often express TFs and signaling molecules, which are markers of stem cells and retinal progenitors (Nes, Mitf, Pax6, Six3, Rx, Chx10, and FGF2), when exposed to in vitro conditions [99,100,101,102,103] (Figure 3). During the formation of CBPE-derived clonogenic spheres in vitro, cells of adult mammals, including humans, enter the S-phase, proliferate, and express “stemness” markers, nestin and TF Pax6. In some cases, cells were detected that expressed protein markers of retinal neurons [100,102,104,105,106]. Taking into account the fact that CBPE cells share a common origin with RPE cells, being their continuation during the eye development, these results have not become a surprise, but have opened up prospects for the replacement of damaged retinal neurons with autologous convertible CB cells [107] (Figure 3).

In a work on the cultivation of postnatal pig CBPE [108], the cells of spheres forming in the suspension culture expressed key pluripotency genes. Thus, mRNAs for *Klf4*, *Sox2*, and *cMyc* were detected, while the transcripts for *Nanog* and *Oct4* were absent. Differentiation was assessed in vitro after the exposure to laminin and the addition of serum. For transplantation, CBPE-derived spheres were dissociated, labeled with CM-DiI vital dye, and injected subretinally into the eyes of eight week-old allo-recipients. Cells positive for both neuronal and RPE markers could be found among transplanted cells. Large clusters of transplanted cells integrated into the RPE layer and multilayered RPE-like structures positive for RPE65 were observed. Grafted cells were also identified in the NR where 5–10% of them were positive for recoverin, protein kinase C alpha (PKCα), and calbindin [108]. Isolation of human pigment and non-pigment epithelium from CB has demonstrated that, like in other mammals, human CBPE cells are more efficient than cells of non-pigmented layers in generating neurogenic spheres [109].

Studies by Cicero et al., Gualdoni et al., and then by Coles and van der Kooy [110,111,112] have clarified the behavior and phenotype of cells present in CBPE-derived cellular spheres in vitro. They have shown that these cells exhibit certain properties of both stem and epithelial pigment cells, in which, in addition to the pigment, there is also the interdigitation of plasmalemma and the epithelial pattern of contacts. Nevertheless, despite the observed proliferation, the partial loss of pigment, and the initiation of conversion to a neural phenotype detected using neuronal markers, no differentiation of retinal cell types occurred in CBPE-derived spheres. Apparently, the differentiation required other, permissive for this, conditions in vitro. Currently, researchers tend to conclude that the adult human CB most likely does not contain bona fide neural stem cells but rather consists of a population of epithelial cells which display a remarkable plasticity in vitro reflecting their neuroepithelial developmental origin [87,113]. It should be noted again that the combination of some behavioral, morphological, and molecular properties of progenitors and differentiated pigmented cells in CBPE during reprogramming is a feature characteristic also of other pigment epithelia of the eye considered in the review. This largely determines their potency to produce cells of retinal phenotypes or to return to the original differentiation.

When studying the regulators of CBPE cells’ conversion under in vitro conditions, it is still poorly understood where their potencies to manifest neural/retinal differentiation properties are fulfilled. Cells have been shown to initially express molecules involved in the Notch signaling pathway. A disturbance in the work of this pathway blocks the neurogenic sphere formation [114,115]. A recently published study has revealed the role of this signaling pathway in CB development [116]. It has been reported that the Notch signaling in CB maintains the vitreous, as well as intraocular, pressure and eye structures by regulating the CB morphogenesis, aqueous humor secretion, and vitreous protein expression. Notch 3 functions redundantly with Notch 2, driving the CB morphogenesis by regulating the expression of adhesion molecule Nectin1. Available data indicate the role of the Notch signaling not only in controlling the CB cell differentiation, but also, as a result, in the key functions of this eye structure, whose disorders form a basis of, e.g., glaucoma [116]. The similarity in the work of this signaling pathway in development and in CB cell-type conversion once again confirms the rule about the involvement of developmental regulatory mechanisms in the cell-type switching of the eye’s pigment epithelium. The involvement of a signaling pathway, including the receptor tyrosine kinase c-Kit and its ligand stem cell factor (SCF), is also assumed to occur along and in interaction with the Notch signaling. As has been shown, these components are able to maintain not only the proliferative activity of cells in CBPE-derived cell spheres, but also the differentiation of their cells along the retinal pathway [114,115].

In addition to TFs and molecular links of regulatory signaling pathways, the epigenetic regulators of CB-derived cells have also been preliminarily studied. Using the material of human cadaveric eyes, Jasty and Krishnakumar [117] analyzed DNA methylation and histone methylation: H3K4me3 and H3K27me3 in a CB-derived lineage of committed progenitors to terminally differentiated cells in vitro. The results showed that several promoters, including pluripotency and lineage-specific genes, become methylated in the differentiating cell population. The authors suggested that methylation may repress the pluripotency, earlier observed in vitro, in this differentiating population. They found also the bivalent modifications involved in the process of differentiation of stem/progenitor cells [117]. Therefore, CB-derived progenitor cells and their progeny that have undergone differentiation into retinal neurons/glial cells show that specific DNA methylation and histone modifications are involved in the gene expression reprogramming during their conversion in vitro. The current epigenetic studies on the conversion of CB- and CBPE-derived cells have implications for understanding not only this process, but also a wide range of pathogenic processes, since the aberrant epigenetic regulation is known to often underlie them [118,119,120].

There is no available information in the literature about the conversion of cells of CB and its pigment layer into any other phenotypes in the spectrum of pathologies, except for neoplastic changes [82,121,122,123]. However, these, as well as changes in the cases of other pigment epithelia, are beyond discussion of the present review.

Apart from tumor formation in CB, the competence of these cells to convert the phenotype in vivo and in vitro is limited to the neural/retinal pathway. Despite the existing similarity between CBPE and RPE, and also the involvement of mesenchyme in the CB development [124], there is no evidence of CBPE cell conversion towards mesenchymal phenotype in the literature. Nevertheless, CB epithelial cells have been reported to exhibit a higher reprogramming efficiency to iPSC than fibroblasts and have a reduced exogeneous requirement. It is expected that the enhanced reprogramming efficiency may be due to increased basal levels of Sox2 in CB cells [125].

Thus, the potencies to the cell-type conversion of CBPE in mammals and humans are based on the presence of a molecular genetic context characteristic of progenitor neuroepithelial cells against the background of a specialization-providing context in the tissue [28]. The former one becomes expressed mainly in conditions permissive for it in vitro, along with the retention and maintenance of some traits of the original CBPE cell phenotype. The cause of the combination of two, seemingly mutually exclusive states, as well as the epigenome’s permission for this condition, may be associated with (1) the late timing of development of this eye’s region relative to the equatorial and central regions of the posterior eye wall, (2) its border position between the posterior and anterior parts of the eye, (3) and also with the molecular specifics of the regulation of this eye region development [87].

## 4. Cell-Type Conversion of the Retinal Pigment Epithelial (RPE) Cells

### 4.1. RPE Cell-Type Conversion as a Basis of NR Regeneration in Amphibians and Birds

Retinal pigment epithelium (RPE) in vertebrates and humans have been studied to a greater extent than other pigment epithelia of the eye as regards cell functions, differentiation, its changes during regeneration and retinal pathologies, and cell transplantation (see, e.g., reviews [17,126,127,128,129]). This is due to the fact that many severe eye disorders (e.g., age-related macular dystrophy (AMD), proliferative vitreoretinopathy (PVR), and *retinitis pigmentosa* (RP)), and other types of retinal degenerations are associated with disturbances in the RPE layer, changes in the phenotype and behavior of its cells, and, as a result, a disturbance of topological, trophic, regulatory, and functional relationships with retinal photoreceptors. Furthermore, some amphibian species and avian embryos manifest the ability to regenerate the retina through RPE cells conversion up to the formation of a new NR at the site of the surgically removed original one.

In adult vertebrates, RPE is an epithelial monolayer of pigmented, polarized, and specialized cells. RPE, lining the NR, is externally bounded by the Bruch’s membrane and choroidal coat (choroid), which supplies essential substances and oxygen to the retina [130,131]. Besides the transport of substances from the choroid to NR, RPE performs a number of other, equally important functions. They include protection against oxidative stress, production and isolation of growth factors, and storage and metabolism of vitamin A derivatives [126,130,131]. The major RPE function is phagocytosis of the outer segments of photoreceptors, their digestion by lysosomes, and retinoid exchange, i.e., providing the processes necessary for light perception.

**Figure 4:**
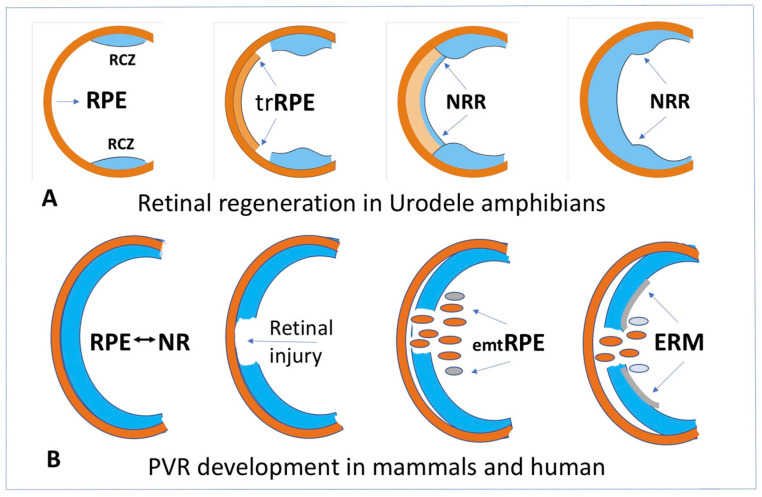
Cell-type conversion in retinal pigment epithelium during neural retina regeneration in amphibia (**A**) and proliferative retinopathy in mammals (**B**). RPE—retinal pigment epithelium; RCZ—retinal ciliary zone; trRPE—transdifferentiating cells of RPE; NR—neural retina; NRR—neural retina regenerate; emtRPE—epithelial-mesenchymal transition of RPE cells; ERM—epiretinal membrane.

RPE cells in caudate amphibians (Urodela) are the most evident example of a natural conversion into retinal cells in vivo. After experimental damage to the NR, optic nerve transection, and artificial detachment or surgical removal of NR, RPE becomes a source of a new, complete, and functioning retina [132,133,134,135,136,137]. The major events of the process are as follows: the exit of RPE cells from the layer, the loss of initial traits and properties (dedifferentiation), proliferation, and the formation of an intermediate population of amplifying neuroblasts. After having reached a certain number, the cells of the NR anlage exit the reproduction cycle, acquire the retinal neuron and glial phenotypes, and begin performing their specific functions [132,133,134,135,136,137] (Figure 4A).

The system of regulation of the RPE cell-type conversion in Urodela has been comprehensively studied, despite the lack of sufficient information about the genome of these animal species used in laboratories until recently. The NR regeneration in caudate amphibians occurs through the control from TFs, signaling molecules, and epigenetic mechanisms. In studies on the native RPE in newts, it was predictably found that the cells express the genes for melanogenic differentiation and specialization [138,139,140,141]. After a disturbance of interaction between RPE and NR, the genes of the immune response and proto-oncogenes, *c-fos*, *c-myc*, and *c-jun*, are activated first [59]. The expression pattern of developmental homeobox genes *Pax6*, *Prox1*, *Six3*, *Pitx1*, *Pitx2*, along with tissue-specific *RPE65* and *Otx2*, was studied at the onset of RPE cell-type switching and during the NR regeneration process [138,139,141]. The up-regulation of the *Pax6*, *Six3,* and FGF2 growth factor genes was shown to occur along with the inhibition of the expression of *Otx2* and tissue-specific *RPE65* and *CRBP* [136,140,142]. It was also found that at the onset of dedifferentiation of RPE-derived cells, the levels of mRNA *Pax6*, *Prox1*, and *Six3* are lower when compared to those in RPE-derived proliferating neuroblasts. The latter showed the transcriptional activity of the *Ns* gene encoding nucleostemin, a nucleolar protein characteristic of stem cells [143,144]. Under conditions of retinal organotypic culture, *Ns* exhibits co-localization with *fgf2* genes, which suggests their co-action in the regulation of cell proliferation [145]. The pattern of the expression of recoverin (Rec), a specific photoreceptor protein, is described during the RPE-derived NR tissue regeneration and the formation of its photoreceptor layer [146]. In all the above-cited studies, the expression patterns of marker and regulatory molecules were found to be similar in the development and regeneration of the retina. The obtained results also indicated the presence of certain components of the molecular genetic profile characteristic of retinal development, along with genes responsible for RPE cells’ specialization, in the native and dedifferentiating RPE cells of mature Urodela [16,28].

Special attention is paid to the period of RPE cells dedifferentiation, the time of choosing further development along the neural pathway. Experiments on isolated cells using quantitative PCR have demonstrated that the first daughter RPE cells at the beginning of retinectomy-induced proliferation manifest expression of the pluripotency genes (*c-Myc*, *Klf4*, and *Sox2*) and, along with them, the developmental genes (*Mitf* and *Pax6*) [147]. The expression of another neural stem cell marker, Musashi-1, has been revealed for the same period [148]. These data, when considered together, suggest the presence of stemness traits in newt RPE cells at the reprogramming stage.

The study by Casco-Robles et al. [149] is significant for understanding the regulation of choice of differentiation pathways for convertible RPE cells. By knocking out the *Pax6* gene in larval newts *Cynops pyrrhogaster* using the Cre-lox technology, it was found that the lack of expression of this master gene subsequently blocks the NR regeneration and induces the RPE cells’ conversion along the mesenchymal pathway. In this case, mature newt RPE cells, while leaving the layer, form multicellular aggregates where the marker proteins of myofibroblasts are expressed: alpha-smooth muscle actin (α-SMA), vimentin (Vim), and N-cadherin (N-Cad). As described below, this is the choice made by human RPE cells, which constitutes the basis for PVR and other diseases associated with RPE mesenchymal transformation [18,19,20]. Casco-Robles et al. [149] suggest that the molecular mechanism used by Urodela for the NR regeneration has evolved in such a way that its modification in mammals has formed the basis for RPE-dependent retinal pathologies. It is worth noting that RPE cells make the choice of the cell-type conversion vector early, shortly after the cells’ leaving the RPE layer, which normally restrains any transformations of the RPE cells’ phenotype.

Understanding the regulation from the cell microenvironment is a clue to the directed alteration of RPE cells’ behavior for the purpose of repairing the retina and/or inhibiting pathological conditions of RPE. The microenvironment factors during the retina-oriented RPE cell-type conversion are largely similar in their range and pattern to the developmental factors. It is also known that in response to the disjunction of NR and RPE, the production/activation/secretion of these factors occurs in NR, RPE, and also in the surrounding tissues of the eye [150,151].

Among the signaling cascades involved in the NR regeneration control in Urodela, special attention is paid to the Fgf, Bmp, Wnt, Shh, and Notch signaling [152,153,154,155,156]. The role of fibroblast growth factor (FGF2), which is a key one for the successful NR regeneration in other animals [157,158,159], has been studied most comprehensively in newts [145,154,160,161]. It was found that, shortly after removing the retina, the *fgf2* genes are down-regulated in the regenerate, which indicates inhibition of FGF2 signals before the initiation of RPE cells’ proliferation. According to these data obtained on a *Pl. waltli* model and information for other Urodela species, FGF2 is not the primary trigger of RPE conversion. The increase in FGF2 expression is associated with a later (~by 1 week) phase of proliferative activity in the forming of an RPE-derived NR anlage. The major sources of FGF2 signals are assumed to be the choroidal coat [162,163], cells of the ciliary marginal zone (CMZ) and Müller cells, and also RPE [161]. The involvement and dynamics of the Notch signal pathway expression during NR regeneration were also studied in newts. The introduction of its blocker (DAPT) was found to result in premature maturation of neurons in the NR regenerate [152,153].

The epigenetic regulation of the RPE cell-type conversion process in Urodela requires further study. Only the first steps have been taken, with a focus on chromatin remodeling [17,164]. Recently, in a study of changes in the epigenetic landscape in mouse RPE cells, an assumption has been made that the expression of “pioneer” TFs in amphibians, as well as the demethylation of regulatory elements of photoreceptor genes, is possible in the RPE cell-type conversion [165]. It is likely that, in sexually mature, paedomorphic newts that initially have juvenile properties [166], the reduction in the level of differentiation (necessary for the conversion) with the involvement of signal–response enhancers, including epigenetic ones, does not require large-scale modifications of gene expression.

The potencies of RPE cells as a source for retinal regeneration through conversion were found in an acaudate amphibian, the frog *X. laevis* [167,168]. The key regulators of the NR regeneration studied in frogs are TFs and components of signaling pathways. FGF2 has been shown to accelerate the RPE cell-type conversion both in vitro and in vivo [168,169]. After retinectomy, FGF2 activates the MAPK pathway, thus, providing the proliferation of RPE cells. In addition, the factor acts as a promoter of differentiation of RPE-derived proliferating retinal progenitors into new retinal cells and is necessary for maintaining Pax6 expression [170]. Inhibition of the MAPK pathway significantly decreases the amount of retina regenerated [168]. It was tested also whether the up-regulation of matrix metalloproteinases (Mmps) triggers the retinal regeneration [171]. In a tissue culture, soon after the retinal removal, the Mmps expression increased in the RPE cells and corresponded to their migration from the choroid. In parallel, IL-1β and TNF-α were up-regulated both in vivo and in vitro. The results suggested that inflammatory cytokines triggered the Mmps’ upregulation. The role of TFs in the specification of NR progenitors during regeneration in frogs was studied using the example of the *rax* gene, whose expression is up-regulated in RPE-derived neuroblasts, while the *rax* knock-down impairs retinal cell types in retina regeneration in tadpoles [172,173]. Hothem et al. [174] and Martinez-De Luna et al. [175] reported about the necessity of the retinal homeobox Rx gene expression for the retinal regeneration in pre-metamorphic *X. laevis*.

The model of retinal regeneration in tadpoles and adult *X. laevis* is currently very promising to be used in studies. Due to the good knowledge of the genome of these animals, the feasibility of using new techniques and approaches for the study of NR regeneration and for testing strategies aimed at its promoting is under discussion now [176,177]. In particular, *X. laevis* is suggested to be used to study the molecular mechanisms responsible for the reactivation of expression of “developmental genes”, which constitute a basis of the process. By postulating their evolutionary conservativeness and similar activation mechanisms, Suzuki and Ochi [178] assign an important role to the signal–response enhancers of gene expression, including also the epigenetic modification of DNA and histones.

The process of retinal regeneration through RPE cell-type conversion in chicks was discovered by Coulombre J.L. and Coulombre A.J. [42]. The regeneration occurs in the early stages of development (up to E4–E4.5); as a result, a new NR with an inverted structure (with photoreceptors facing the eye cavity, rather than the RPE) is formed [179]. As regards the immune system, the C3a complement has been shown as an inducer of NR regeneration through the activation of the STAT3 transcription regulator, which, in turn, activates the damage response factors IL-6, IL-8, and TNF. This eventually leads to the regulation of the Wnt2b signaling pathway genes, and also to the expression of the *Six3* and *Sox2* genes characteristic of retinal progenitors [180]. A study by Zhu et al. [181] on the expression of β-catenin, a well-known coordinator of cell–cell adhesion and gene transcription, showed that nuclear β-catenin positive cells remain in the RPE in response to injury. These cells were BrdU-negative and p27-positive, which suggested that nuclear β-catenin prevents them from entering the cell cycle. In the presence of FGF2, the RPE cells, while dedifferentiating and proliferating, lost the expression of nuclear β-catenin. A retinectomy, followed by disruption of active β-catenin using a signaling inhibitor (XAV939), induced regeneration from RPE in the absence of FGF2. Thus, inactivation of β-catenin seems a necessary pre-requisite for the NR regeneration in chicks [181]. At the stage of the proliferative phase of RPE cells’ conversion in birds, as in other animals, FGF2 activates the cell multiplication and, as a result, the subsequent histogenesis of the NR regenerate. In the layer of amplifying neuroblasts, there is an expression of proneural markers, which are TFs (Pax6 and Chx10) regulated by the FGF2-FGFR/MEK/Erk signaling cascade [182,183]. In chicks, as well as in newts, a down-regulation of RPE65 specific protein and the TF Mitf, which are participants in the RPE specialization of eye development, occurs during the RPE cells’ conversion [182]. These data were supplemented by the results showing the expression of the factors klf4, c-myc, and lin-28, which are markers of cell pluripotency. The RNA binding protein lin-28 is a target for the action of FGF2, and its overexpression results in the RPE cell-type conversion in the absence of FGF2 as well [184]. The use of the model of NR regeneration by RPE in chicks has also revealed the involvement of Shh [185], BMP, and Wnt signaling cascades [186]. It is worth noting that at the stage of two prospective layers of the retina anlage (RPE and NR) at the optic cup stages in chicken embryo, a low concentration of BMP leads to the RPE cell-type conversion in NR, while a high concentration, on the contrary, leads to the conversion of embryonic NR cells into pigmented RPE cells [186]. A study of the loss of function showed that the NR conversion into RPE requires both BMP and Wnt signaling. Steinfeld et al. [186] suggest that manipulations with these key signaling pathways may contribute to the development of efficient standardized protocols for RPE and NR generation required to create cell replacement therapies.

To characterize the mechanisms underlying the RPE cell conversion in the chicken embryo, RNA was extracted by laser capture microdissection from an intact RPE tissue, tissues at 6 h post retinectomy, and cells converted in the presence of FGF2 [183]. Using RNA-seq, the authors observed the repression of genes related to cell cycle progression in the early-stage converting RPE, and also the up-regulation of injury-associated genes. In contrast, the RPE, converted in a FGF2-dependent manner, was enriched in MAPK-responsive genes and retina development factors, thus, confirming that FGF2 and the downstream MAPK cascade are the main drivers of the embryonic chick RPE reprogramming. Tangeman et al. [183] made an attempt to understand the role that the mechanisms responsible for epithelial–mesenchymal transition (EMT), which is a RPE cell-type conversion underlying retinal pathologies in mammals, can play in the conversion of chicken embryo RPE cells. Since the EMT program is accompanied by an extensive, coordinated regulation of ECM regulators (see below), the major focus is on the regulators of ECM state and composition [183].

As has been repeatedly mentioned, a switch of cell types is accompanied by resets of the epigenetic landscape that drive shifts in transcriptional programs and, eventually, in cell identity. Currently, the whole genome bisulfite sequencing (WGBS) analysis of DNA methylation in parallel with the up-to-date sequencing technology is used to study them. These methods were applied to describe global epigenetic patterns and fluctuations during the development of the chicken embryo retina. This allowed for comparing the global changes in DNA methylation to differential gene expression during chick developmental retinogenesis [187]. Luz-Madrigal et al. [188] applied the same approach to the study of the NR regeneration process in a chicken embryo, where RPE prior to injury and during different stages of RPE reprogramming were considered. The changes in the expression of genes associated with epigenetic modifications during RPE reprogramming, the dynamic changes in histone marks associated with bivalent chromatin (H3K27me3/H3K4me3), and intermediates of the process of DNA demethylation, including 5hmC and 5caC, were studied. The data confirmed the extensive rearrangements of DNA methylation patterns, including differentially methylated regions found at promoters of genes associated with chromatin organization and FGF2 production. The role of FGF2, according to the authors of the study, consists of supporting these dynamic modifications that are further sustained in the commitment to form a new NR. In general, the findings reveal the active DNA demethylation as an important process that may be applied to remove epigenetic barriers in order to regenerate retina in mammals [188]. Thus, in studies based on animal models such as amphibians and birds that demonstrate NR regeneration through the RPE cell-type conversion, a significant similarity can be found between the molecular mechanisms that accompany the process, including regulatory ones, and those operating during the normal development of the eye’s retina. The similarity is found both in the conversion triggers and in the expression of conserved, “developmental” TFs, the expression of genes from the “pluripotency induction factors” group, and also in the set and up-regulation of expression in a number of signaling networks. The competence of RPE cells to the cell-type conversion in amphibians and birds along the retinal pathway and its implementation are based on extracellular and intracellular specifics of cell behavior regulation, including epigenetic ones. The same is applicable to mammals and humans, as shown below.

### 4.2. RPE Cell-Type Conversion In Vitro

Over the past 20 years, extensive information has accumulated, indicating the RPE cell-type plasticity and a capability of conversion under in vitro conditions. RPE cells of adult rodents [189] and human cells in vitro [190,191,192,193], exposed to morphogens and growth factors in the medium, demonstrate a decrease in the level of differentiation, proliferation, and, in some cases, production of neurons (Figure 3B). In the study by Engelgardt et al. [189], RPE cells of adult rats in vitro were found to express progenitor markers: the cytoskeleton protein Nestin and the RNA-binding protein Musashi1 (Msi). Subsequently, such cells can produce cells of the pro-neural phenotype and express their markers: doublecortin (DCX) and β-III tubulin [189]. The use of a mouse embryo RPE cell model showed the inhibition of such conversion through blocking activin, a signaling protein of the TGFβ family [194]. In the report of Chen et al. [195], the cultivated mouse RPE cell-derived “neurospheres” contained reprogrammed cells that could either return to the original RPE phenotype or acquire photoreceptor differentiation. In the case of transplantation of such spheroids subretinally into animals’ eyes with simulated retinal degeneration, they showed the ability to be integrated in the structure of the injured NR and replace its lost cells.

As evidenced by studies on isolated human eye RPE cells and their immortalized lines, these cells, while dedifferentiating in vitro, down-regulate the expression of the specific protein RPE65 and express the *OCT4*, *NANOG*, *KLF4*, *OTX2*, *PAX6*, and *NESTIN* genes, which represent markers of low differentiation [190,191,196,197]. In some cases, this was followed by the expression of marker proteins of more advanced neuronal differentiation: tyrosine hydroxylase (TH) and neurofilament proteins (NFs) [191]. Using human RPE cells in vitro, in was found also that FGF2 is capable of stimulating cell conversion along the neural pathway [198]. Of equivalent importance are also the data showing the possibility to prevent human RPE cells and their lines from transformation and stabilize them in the state of initial differentiation while being exposed to a number of factors in vitro. Transcriptome studies on such cells have revealed the expression of surface and functioning markers similar to those of native human RPE cells [199,200,201,202]. When analyzing the behavior of human RPE cells in vitro, Burke J.M. [9] concluded that their phenotype largely depends on the regulation along the Wnt/β-catenin pathway. Furthermore, this signaling was shown to play a role in regulating the response of RPE cells to oxidative stress [9]. Salero et al. [203] report that human RPE cells in vitro are capable of retaining the original phenotype or producing cells of not only neural differentiation, but also cells expressing markers of mesenchymal derivatives: muscle, adipo-, osteo-, and chondrogenic cells. This occurs when using media that contain stimulatory supplements corresponding to obtaining of each of the differentiations listed. In studies on a human RPE cell line (ARPE-19), the regulatory role of the Wnt signaling pathway was again indicated for the RPE cell-type conversion along the mesenchymal pathway [10]. In general, the data obtained in vitro are the evidence of the initially laid down broad competence of RPE cells in adult mammals and humans, manifested in vitro along the initial epithelial, neural, and mesoderm-associated pathways of differentiation. Taking into account this behavior of mammalian and human RPE cells during in vitro cultivation, some authors [88,203,204] regard RPE (or a certain population of its cells) as stem cells (RPESCs), a priori assuming their presence in human RPE. However, despite the discovered cellular heterogeneity in RPE [205,206], there is no direct evidence of the presence of RPESCs in it.

Molecular intracellular changes in mammalian RPE cells in vitro show some similarities to those occurring in amphibian and avian RPE during in vivo conversion, which indicates that certain links of molecular mechanisms of RPE cell-type conversion were retained in the evolutionary series of vertebrates. In addition, mammalian RPE has properties that are required for the initiation and progress of reprogramming along the retinal pathway, e.g., the proliferative activity or modulation in the production and loss of melanin granules [207]. It has been shown that mammalian RPE cells can proliferate in vivo, though in very limited numbers, mainly on the periphery of the layer [208]. Studies on proliferation of isolated human RPE cells under in vitro conditions have shown that these cells implement the same mechanism of S-phase entry as in the case of proliferation of amphibian and avian RPE cells in vivo (see above), where MAPK and ERK kinases play a key role [209]. On this signaling pathway, the growth factor receptor activates rasGTP-ase, which results in the MAPK/ERK phosphorylation [210]. MAPK/ERK, in turn, up-regulates the expression of transcripts (*c-myc*, *Pax6*, *klf4*, and *Mitf*), which indicates a decrease in the level of RPE cells’ differentiation [211]. However, these potencies are not implemented in vivo either for the regeneration of the RPE layer or for the conversion aimed at NR regeneration. It is obvious that mammalian RPE appears to be poor in the regulatory elements required to control cell division and induction of transdifferentiation along the neuronal pathway [208]. According to Rzhanova et al. [212], terminally differentiated mammalian RPE is rich in epigenetic regulatory mechanisms that fix specific patterns of gene expression.

### 4.3. RPE Cell-Type Conversion In Vivo as a Basis of Retinal Diseases in Mammals and Humans

In mammals, RPE-dependent NR regeneration is extremely limited. The mammalian retina does not regenerate spontaneously after injury or in disease [17,213,214]. An injury results in cell death, loss of the affected neurons and RPE cells, and, in some cases, in RPE cells’ conversion into cells with mesenchymal phenotype. The RPE transdifferentiation along the neural pathway was observed in vivo, in embryos of a mutant mouse line after elimination of the RPE-specific TFs Mitf or the Wnt signaling effector β-catenin [215]. The same could be observed in microphthalmia (*mi*/*mi*) mice [216]. Other data suggests that the mammalian RPE has lost its latent capacity to generate retinal cells in vivo.

**Figure 5:**
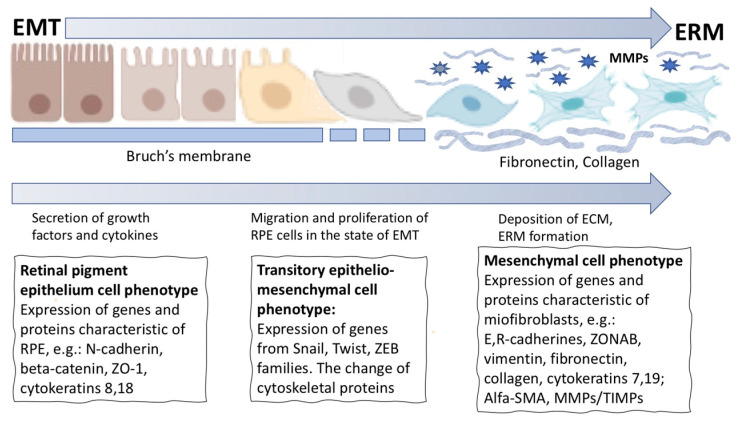
Retinal pigment epithelium cell-type conversion in the mesenchymal direction during the development of proliferative retinopathy (PVR). EMT—epithelial-to-mesenchymal transition; ERM—epiretinal membrane; MMPs—matrix metalloproteinases.

In a case of retinal pathology such as retinal detachment and rupture in mammals and humans in vivo, similarly to that in amphibians, part of the RPE cells at the first stage lose their epithelial properties, leave the layer, migrate, and proliferate. These processes are accompanied by death or conversion of part of the cells with the acquisition of myofibroblast properties (Figure 4B). These cells, when moving beyond RPE and NR and being exposed to the vitreous humor factors and the factors secreted by surrounding tissues, initiate the synthesis of ECM components, proliferate, and become involved in the formation of the epiretinal membrane (ERM) [217,218,219,220] (Figure 4B and Figure 5). RPE cells are the main participants in the ERM formation that play a crucial role in proliferative retinopathy (PVR). Furthermore, fibroblasts and macrophages also make a significant contribution to the ERM formation [20,221]. The formation of ERM and the intrinsic contraction of its cells lead to the tractional forces responsible for the clinical features of PVR. Descriptions of the stages and mechanisms of regulation of this process are available in the literature [19,20,219,222]. Attention to them is associated mainly with PVR, an unwelcome wound healing process of the retina and the cause of approximately 10% of all retinal detachments [151]. PVR with recurrent retinal detachments requires additional surgical interventions and is associated with poor visual recovery. The process of RPE cells’ conversion in PVR is described in terms of epithelial–mesenchymal transition (EMT) or “RPE dysfunction” [223], although the definition “RPE cell-type conversion into mesenchymal phenotype” is also valid (Figure 5). This process occurs not only in PVR, but also in the case of attempts to restore the RPE layer after laser-induced damage [224], and also in proliferative diabetic retinopathy [21] and subretinal fibrosis [225,226]. The latter case, similar to that in PVR, is characterized by proliferation and/or infiltration of RPE cells, glial cells, fibroblasts, myofibroblast-like cells, and macrophages, which, when interacting with inflammatory cytokines and growth factors, begin to synthesize and remodel the EMC. The above events are the final accompaniment of neovascular age-related macular degeneration (nAMD) [226,227].

A detailed consideration of EMT as a phenomenon is impossible within the framework of this review, due to the broad occurrence of this phenomenon in animals and humans: EMT is the basis for laying down organs in development and regeneration in invertebrates and vertebrates, cell reprogramming in fibrosis and tumor changes, and also metastasizing [228,229,230].

The stability of RPE cell differentiation in the layer is supported by the coordinated interaction of genes with the regulatory network that controls the homeostasis processes and tissue functions. EMT of RPE cells in situ is an aberrant tissue response, an attempt to restore it that ends with ERM formation [219,231]. Specifics of this response of mammalian RPE cells are manifested as the loss of polarity, destruction of cell–cell junctional complexes, including adhering junctions and tight junctions, and also detachment from the Bruch membrane lining the RPE. These are followed by the rearrangement of the cytoskeleton and the acquisition of properties similar to those of mesenchymal cells [232,233]. The major event of the rearrangement of cell contacts in RPE during EMT is a change in the cadherin expression patterns [234,235]: inhibition of the N-cadherin (CDH2) expression and switching to the expression of cadherins E (CDH1) and R (CDH3) [236,237]. The cytoplasmic domain of cadherins regulates interactions between cadherins and catenins, including β-catenin, which is able to regulate TF expression and cell proliferation. In addition to cell–cell contacts, the fate of RPE cells in proliferative diseases of the human retina is largely determined by the state of the intercellular matrix [238]. A substantial role is played not only by cadherins and related catenins, but also by the polypeptide zonula occludens-1 (ZO-1), which is present at sites of cell–cell adhesion. When the phenotype of mouse RPE cells changed from pigment epithelial to fibroblast-like, the activity of ZONAB (ZO-1-associated nucleic acid binding protein) was shown to increase and that of ZO-1 to decrease [239], which indicates the role of ZO-1/ZONAB pathway in the process of mammalian RPE conversion. During EMT in RPE in vivo, there is a decrease in the expression of desmoplakin, intermediate filament proteins, and other participants of desmosome formation [240].

The contractile protein α-SMA (alpha-smooth muscle actin), which provides cell mobility of RPE, is an intracellular protein considered, first of all, as a marker of the epithelial–mesenchymal differentiation. In addition, the cytoskeletal protein vimentin (Vim) also plays an important role in stabilizing the structure of migrating cells. During EMT, the pattern of cytokeratin expression changes [240], the Vim and α-SMA expression increases, and ECM proteins, including collagen and fibronectin (FN), begin to be deposited [241]. FN, a marker protein of fibrosis produced by cells, is necessary for the accumulation of microfibrils during the ERM formation [20,242].

The molecular–genetic bases of EMT of RPE cells, including changes in gene expression and its regulation, remain incompletely understood. However, as in other examples of RPE cell-type conversions in animals, it is evident that these ones involve changes in the expression of functionally significant genes controlled by specific TFs, regulatory signaling systems, and epigenetic factors. The EMT process is accompanied by a change in the expression pattern of TFs of the snail superfamily, as well as in slugs, including ZEB1/2, TWIST, and GSC [227,243,244], and also a number of other TFs accompanying EMT in fibrotic and tumor transformations [245,246]. TFs play a major role in the initiation of E-cadherin expression, which is the key mechanism of EMT. Significant TFs in the regulation of the RPE cells’ phenotype were identified using bioinformatic and biochemical analysis methods [247]. Specific elements that are responsible for transcription during binding to TFs (regulators of EMT in RPE) and, accordingly, are potential targets for the prevention and treatment of pathologies associated with proliferating RPE cells were identified in the promoters of some genes. These include Oct-1, hepatocyte nuclear factor 1 (HNF-1), nuclear transcription factor GATA-1, SMAD3, transcription factor E (TFE), interferon regulatory factor-1 (IRF), HNF3alpha, E2F, CDP, SP3, homeobox-containing gene NKX3A, sterol regulatory element-binding protein-1 (SREBP-1), lymphocyte enhancer-binding factor-1 (LEF-1), etc. [227,247].

RPE cells obtained from human embryonic stem cells (hESCs), embryonic RPE, and the ARPE19 cell line are often used as models to study the molecular mechanisms of RPE cell-type conversion by EMT [223,248,249,250]. It was found that the FOXM1 proto-oncogene is a participant of EMT in the above-noted cells [251]. Its overexpression “enhances” the epithelial phenotype of RPE, as evidenced by the level of expression of the marker, the premelanosomal protein PMEL17. FOXM1 is also known to directly regulate the proliferation of RPE cells due to its association with genes that are cell cycle regulators [252]. The knockdown of FOXM1 using siRNA leads to a decrease in the expression of positive cell cycle regulators (CDC5L, CDK12, and FZR1) and an increase in the expression of the cell cycle inhibitor CDKN1A [253,254]. The EMT mechanism also includes the action of FOXM1 in modulating the expression of the signaling proteins BMP7 and Wnt5B. The exogenous recombinant Wnt5B is known to significantly reduce the expression of epithelial markers when the epithelial phenotype changes towards the mesenchymal one [243]. As regards the gene expression in PVR, the role of microRNAs (miRNAs), small, endogenous, noncoding RNAs that negatively regulate gene expression within cell types, has been preliminarily studied. In the work of Toro et al. [255], a total of 754 miRNAs were subjected to real-time PCR expression profiling in order to identify the differentially expressed miRNAs in the vitreous humor of patients diagnosed as having primary retinal detachment and PVR progression. The study showed that the expression of miR-143-3p, miR-224-5p, miR-361-5p, miR-452-5p, miR-486-3p, and miR-891a-5p increased with the worsening of PVR grading and could be considered good candidates for PVR biomarkers [255].

Both growth factors and inflammatory factors are the mediators of the EMT process in PVR. The list of important EMT regulators includes TGF-β (master regulator EMT), PDGF, EGF, FGF, VEGF, CTGF, IGF2, IL-1a,β, IL-2,3,6,8, TNF-a, and also the adhesion factors ICAM-1, etc. Among them, TGF-β, TNF-a, PDGF, IL-6, and IL-8 are considered to be particularly important in this process (reviews: [227,256,257]). The occurrence of ERM in PVR is associated with the activation of the most comprehensively studied TGF-β and TNF-a signaling pathways that are key ones in this process [258,259,260]. These factors synergistically activate the EMT program in adult human RPE cells. Increased levels of TGF-β [259] and TNF-a [261] were recorded from the vitreous body of patients with PVR and observed to correlate with severity of this disease.

Attempts are also made to compose a map of related epigenetic and transcriptional changes in RPE in normal conditions and in the case of EMT [256,262]. It has been shown that the most pronounced epigenomic changes accompanying EMT are associated with the increase in active chromatin tags on many elements of enhancers of the presumptive TF binding targets, which, in turn, are key regulators of the process. The gene regulation landscape accompanying EMT has been identified through comparing the data of the study of the RPE epigenome and transcriptome in normal conditions and after treatment with the TGF-β1 and TNF-a factors. Among the strategies for inhibiting signaling pathways involved in RPE pathology, the modulation of RhoA/Rho-kinase, Smad, or MAPK signaling is also considered [263,264,265]. In RPE cells derived from human pluripotent stem cells and induced towards EMT, the TGF-β pathway was inhibited after treatment with nicotinamide (NAM), a derivative of vitamin B_3_ [266]. The exposure to NAM enhances the epithelial phenotype and prevents EMT, which allows considering this approach as a method of therapy for patients with ERM [265]. Other approaches suggest blocking the expression of one of the receptors of the canonical TGF-β signaling pathway (TGF-β receptor 1) using its inhibitor LY-364947 (LY) to prevent PVR [267].

## 5. Overall Discussion

An attempt to summarize data on the RPE cell conversion along the mesenchymal pathway in vivo, which occurs in mammalian and human retinal pathologies such as PVR, has demonstrated that the main stages of this process are similar to those observed during the RPE cell transdifferentiation along the retinal pathway in vivo, which underlies the NR regeneration in amphibians. Similar features are the initiation of conversion, the displacement of RPE cells from the stabilizing environment (niche), the loss of cell–cell contacts and contacts with surrounding tissues, the remodeling of cell surface and cytoskeleton, the proliferation, migration, and change of metabolome, the genome reprogramming, and, eventually, the consistent acquisition of another phenotype. However, when discussing the molecular and genetic mechanisms of mesenchymal and neural conversion strategies of RPE, we find only minor similarities and substantial differences.

When the conversion is initiated as a result of disjunction in the single functional RPE–NR system, the changes in cadherin/catenin protein compositions of contacts and associated signal cascades are observed to play a similar trigger role. The launch of the molecular “rescue mechanisms”, discussed in detail in the review by Grigoryan E. [150], is also a common feature. The similarity can also be seen in the trigger effect of the components of the immune and circulatory systems [256,268], which, however, differ significantly between lower and higher vertebrates. The development of RPE cell-type conversions along the neural and mesenchymal pathways from the aspect of changes in the molecular genetic internal and external patterns occurs in different ways. These include different dynamic regulatory networks formed by microenvironment factors, TFs, and the epigenome.

As regards changes in the epigenome, its well-known general mechanism, accompanying all stages of conversion and very preliminarily studied for the examples given in the review, is the chromatin remodeling and the active DNA demethylation along with the initiation of “pioneer” factors’ expression. However, it should be noted that little data explaining the epigenetic regulation of cell-type conversions of pigment epithelia of the eye in lower vertebrates are available to date. So far, it can only be assumed that, in these animals, the enhancers of genes responsible for reprogramming are not strictly silenced by epigenetic modifications. All these interdependent regulatory factors (TFs, signaling pathways, and epigenomic changes), exhibiting certain evolutionary conservatism, gather in different regulatory networks with different RPE conversion strategies.

Recently, Hoang et al. [269] have attempted to summarize information about the operation of such networks on the Müller cell conversion model (another well-known potential source of NR regeneration, [270]) from studies on zebrafish, chicks, and mice. Using cross-species RNA-seq and ATAC-seq, and also the comparative bioinformatic analysis, the authors of the study aimed to identify the evolutionarily conserved and species-specific gene networks controlling Müller glia quiescence, reactivity, and neurogenesis. Authors [269] suggested that such an approach in the future would make it possible to directionally change the targeting gene regulatory networks that repress neurogenic competence.

A return to the active expression of many developmental genes was revealed in studies on amphibian IPE and RPE cells during in vivo conversions along the lens and retinal pathways that led to the regeneration of lens (IPE) and NR (RPE). The studies also show that these genes, involved in regeneration, are frequently conserved among vertebrates. The same pattern—up-regulation of the expression of “developmental” genes—is found in the processes of IPE, CBPE, and RPE cell-type conversions of mammalian and human cells along the neural/retinal pathway in vitro. Besides “developmental” genes, the expression of TFs, the known inducers of pluripotency (OSKM) [4], was observed in the process of neuronal conversion in IPE, CBPE, and RPE of vertebrates (including humans) regardless of their taxonomic affiliation. Of particular note is the similarity of the active key signaling pathways where FGF2 plays a dominant role during the IPE, CBPE, and RPE cell-type conversions along the neural and lens pathways. The canonical mechanism of cell entry into the proliferative phase, where MAPK/ERK is the FGF2-downstream signaling cascade, is also uniform. These molecular genetic mechanisms are not observed in cases of RPE conversion along the mesenchymal pathway in vivo. As mentioned above, there is an opinion [149] that the molecular mechanism used by amphibians for the NR regeneration has evolved and significantly modified in such a way that eventually formed a basis of RPE-dependent retinal pathologies in mammals and humans.

The pigment epithelia considered in the article—IPE, CBPE, and RPE—have a common origin from neuroepithelium that originates from the cephalic ectoderm [211,271]. For this reason, the potential of cells of these eye tissues to be converted along the neural and lens pathways is not unexpected. The manifestation of the competence of mammalian and human RPE cells to convert along the mesenchymal pathway under experimental conditions or in the case of eye damage/pathology requires a different explanation. One of them, in addition to the effects of the above-mentioned various specific signaling systems, is a deeper differentiation of RPE in its central region, where damages are more often detected when compared to the eye “periphery”, NR ciliary zone, and CB, regions that form later in the eye development. According to data in the literature available to date, CBPE, while remaining a relatively young, “transitory” compartment of the eye [211,271], does not demonstrate conversion along the mesenchymal pathway either in vivo or in vitro. Another explanation is associated with the taxonomic position of mammals and humans. The events that occur in vivo in the RPE of humans in above-discussed diseases are specific to this taxon. In animals related to it, tissue regeneration in most cases has the pattern of scarring, aimed at rapid wound healing. It is assumed that in the course of evolution, the longer in time tissue regeneration in poikilothermic animals and rapid scarring in homeothermic animals were in a competitive relationship. As a result, the latter animals have acquired methods for rapid protection against infections with a more mature immune system and rapid healing through scarring, while, simultaneously, losing the ability to form tissue and whole structures de novo [272]. At last, a cause of the RPE cell conversion along the mesenchymal pathway may be the EMT phenomenon proper, which is an aberrant response that can affect epithelia of various origins, including RPE, undergoing a drastic change in regulatory programs in response to the changes in the signaling environment [262,273].

## 6. Conclusions

In the present review, we have considered the examples of cell-type conversions of pigment epithelia of the eye (IPE, CBPE, and RPE) in various animals and in humans, in the case of pathologies associated with the conversion into mesenchymal cell phenotypes. Studies conducted on animal models, as well as in vitro, currently provide a general idea of how the conversion is carried out, and also what determines and controls it. The pathway of phenotypic switching inherent in all the pigment epithelia of the eye is neural/retinal. This type of conversion underlying the retinal regeneration is found in amphibians and birds in vivo. In mammals and humans, the conversion along the retinal pathway occurs only and exclusively in vitro, with targeted stimulation of the process. Amphibian IPE also shows the ability to convert into lens cells, being a source of lens regeneration in vivo in some species; in vitro it can be reprogrammed into both the neural and lens cell phenotypes. Mammalian and human RPE in vivo demonstrates conversion along the mesenchymal pathway, leading to PVR and other eye pathologies associated with fibrosis. However, RPE implements a broader range of potencies in vitro, converting additionally into neural/retinal cell types.

Molecular genetic studies of conversion processes have made it possible to identify the mechanisms of their implementation, the causes and the timing of selection of which being a particular strategy for changes in cell differentiation and their reprogramming pathways. It also became possible to determine the molecular and genetic bases of conversion processes and their regulation, including species-specific molecular context, differential expression of transcription factors and signaling pathways, and substantial epigenomic changes as well. These bases were formed during phylogeny and develop each time into a pattern typical of each taxon during ontogeny. This pattern, while demonstrating some evolutionary conservatism, has specifics that determine the strategy (strategies) and the conversion outcome: manifestation of regenerative capabilities or progression of pathology of the retina and other eye tissues.

The key molecular participants in the implementation of inherent competencies can be identified on animal models in vivo and also on in vitro models that allow for experimentally reproducing the cell-type switching processes by various biotechnological approaches and methods. Stimulation of the regenerative capabilities of eye’s pigment epithelia, i.e., their conversion along the retinal pathway, requires the conditions that stimulate proliferative activity, restore the expression pattern of developmental genes and multipotency genes (i.e., “rejuvenation” conditions), and also provide control from the microenvironment for further development of emerging cell lines along the desired pathway. Some of these conditions are known and can be reproduced in vitro and, to a limited extent, in vivo, but, nevertheless, much remains to be learned. Thus, the miRNA-based regulatory mechanisms of cell response to injury and the influence of inflammatory signals on the regenerative process are still far from being fully understood. There is also a need for a cross-species comparison of the eye pigment epithelium transcriptomes using single-cell technologies. It is also necessary to establish new strategies for promoting both the proliferative and neurogenic potentials with a strict prevention of unwanted transformations. It has become evident that the loss of regenerative potential and the frequent pathological changes in tissues lead to inactivation of certain genes as the organism ages. For this reason, further comparisons of the epigenetic controls over the expression of regeneration-related enhancers between different taxa and throughout the lifespan are required.

Views on the phenomenon of eye pigment epithelial tissues conversion urgently need further development on the basis of accumulated data and using novel technologies. This may provide a clue to understanding the relatedness or heterogeneity of the cell-type switching mechanisms leading to positive (regeneration) and negative (pathology) outcomes in the same pigment epithelial tissues in different groups of vertebrates and in humans. This knowledge can eventually be applied in regenerative medicine to prevent degenerative disturbances in damaged or diseased human retina and to create adequate and safe conditions for its regeneration. The key molecules and links in the mechanisms of cell-type conversion regulation discovered to date currently allow identification of targets and factors to have an effect on them for the therapeutic prevention of retinal pathologies associated with the proliferation and conversion of RPE and other pigment tissues of the eye.

## Data Availability

Not applicable.
